# Pars Plana Vitrectomy for Vitreomacular Traction Resulting in Persistent Postoperative Loculated Foveal Subretinal Fluid

**DOI:** 10.1177/24741264231176137

**Published:** 2023-06-06

**Authors:** Darcie S. Wilson, Ashlyn M. Pinto, R. Rishi Gupta

**Affiliations:** 1 Faculty of Medicine, Dalhousie University, Halifax, NS, Canada; 2 Department of Ophthalmology & Visual Sciences, Dalhousie University, Halifax, NS, Canada

**Keywords:** vitreomacular traction, subretinal fluid, pars plana vitrectomy

## Abstract

**Purpose:** To describe a case of postoperative persistent loculated subretinal fluid (SRF) that developed after pars plana vitrectomy (PPV) for vitreomacular traction (VMT) syndrome. **Methods:** A case was analyzed and a literature review performed. **Results:** A healthy 64-year-old man with no significant ocular history presented with persistent, symptomatic VMT. Combined phacoemulsification and PPV, epiretinal membrane and internal limiting membrane peeling, and gas–fluid exchange were performed. Postoperative spectral-domain optical coherence tomography imaging showed loculated foveal SRF. The SRF persisted for 8 months, with minimal change in size and little best-corrected visual acuity improvement. **Conclusions:** Although persistent loculated SRF has been reported after vitrectomy for rhegmatogenous retinal detachment (RD) in high myopia and tractional RD in diabetes, it has not yet been reported postoperatively after PPV for VMT. Studies of the pathophysiology and long-term course of persistent SRF after PPV for VMT are needed to inform management decisions for this rare complication.

## Introduction

Vitreomacular traction (VMT) syndrome is a disorder of the vitreoretinal interface defined by partial vitreous detachment and a posterior hyaloid membrane strongly adhered to the macula, causing anteroposterior traction along the adherent site. The resulting tractional forces have the potential to cause morphologic and functional changes to the macula, including the development of an epiretinal membrane (ERM), cystoid macular edema (CME), full-thickness macular hole (FTMH), and macular detachment.^1^ Although some cases of VMT are asymptomatic, the gradual disruption and elevation of the fovea can cause visual symptoms including decreased vision and metamorphopsia.^1^

VMT can spontaneously resolve, in particular if the disease is grade 1.^2^ Hence, observation is a reasonable initial management option.^3^ Pars plana vitrectomy (PPV) remains the gold standard treatment for persistent and symptomatic VMT that is significantly affecting a patient’s quality of vision. The goal of PPV for VMT is to relieve the tractional forces on the macula and allow for reconstitution of the normal foveal contour and architecture. Postoperative outcomes of PPV for VMT have been extensively described in the literature, with reports of a high success rate in improving macular morphology, metamorphopsia, and best-corrected visual acuity (BCVA).^4,5^

We present a case of a 64-year-old man who had PPV for symptomatic VMT. The case was complicated by postoperative development of persistent loculated foveal subretinal fluid (SRF). To our knowledge, this has not yet been described in the literature.

## Case Report

A 64-year-old man presented with 3 months of progressive metamorphopsia and blurred vision in the right eye, which led to patching of the eye to alleviate symptoms. His medical and ocular histories were otherwise unremarkable.

On examination, the BCVA was 20/70 OD and 20/20 OS. He was mildly myopic with a refraction of −2.00 + 2.25 × 20 and −1.50 + 1.75 × 167, respectively. The intraocular pressure was 12 mm Hg in the right eye and 10 mm Hg in the left eye. The axial length (AL) was 25.05 mm and 25.08 mm, respectively.

**Figure 1.**
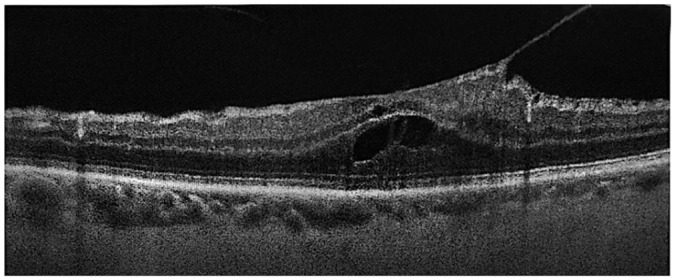
Spectral-domain optical coherence tomography of the right eye preoperatively shows broad vitreomacular traction with an abnormal foveal contour, mild cystoid spaces, and an epiretinal membrane.

Fundoscopic examination of the right eye showed an abnormal foveal light reflex but was otherwise unremarkable; specifically, there were no retinal breaks, preoperative pigment epithelial detachment, or areas of SRF. Spectral-domain optical coherence tomography (SD-OCT) showed broad adhesion from the vitreous with traction distorting the normal foveal contour. SD-OCT showed mild cystoid spaces and an ERM (Figure 1). The patient consented to combined phacoemulsification and vitrectomy surgery.

A DCB00 19.0 D Tecnis intraocular lens (Johnson & Johnson) was implanted in the capsular bag after uneventful phacoemulsification surgery. During the vitrectomy, the posterior hyaloid membrane was extremely adherent to the retina and the fovea appeared to elevate slightly from the retinal pigment epithelium (RPE) during hyaloid separation. This appearance was maintained to the conclusion of surgery. An FTMH was not evident; however, there was concern about the development of a microhole. Therefore, this was treated as an MH case and a membrane peel was indicated.

Anteriorly, there was a very posterior insertion of the vitreous base. Thus, in an effort to avoid iatrogenic breaks, the hyaloid was cautiously elevated only to the midperipheral retina. Indocyanine green was used to stain the internal limiting membrane (ILM); both the ERM and ILM were also found to be extremely adherent. Slowly and progressively, these were peeled in a wide fashion to relieve all traction. During surgery, a fluid–air exchange was performed followed by a gas–air exchange with 18% sulfur hexafluoride. The patient was asked to maintain facedown positioning for 5 days.

**Figure 2.**
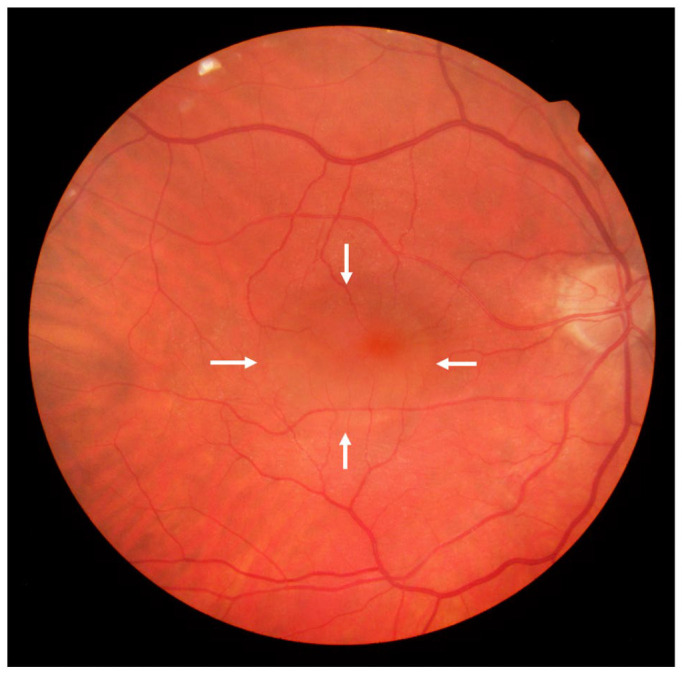
Posterior pole fundus photograph of the right eye 2 months postoperatively shows persistent foveal subretinal fluid (arrows).

**Figure 3.**
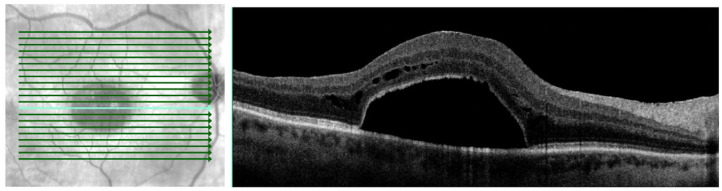
Spectral domain optical coherence tomography of the right eye 2 months postoperatively shows loculated foveal subretinal fluid.

**Figure 4.**
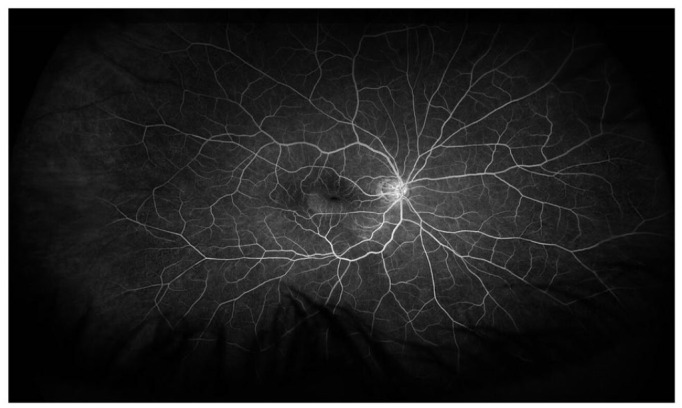
Fluorescein angiography of the right eye 2 months postoperatively shows subretinal fluid and no macular neovascularization.

Two months postoperatively, the BCVA in the right eye was 20/100, with fundoscopic examination showing foveal SRF (Figure 2). Further SD-OCT imaging showed loculated foveal SRF (Figure 3 and Supplemental Video). Multiple scans with dense rasters as well as radial patterns were used to rule out a full-thickness macular microhole. Fluorescein angiography did not show macular neovascularization (Figure 4).

**Figure 5.**
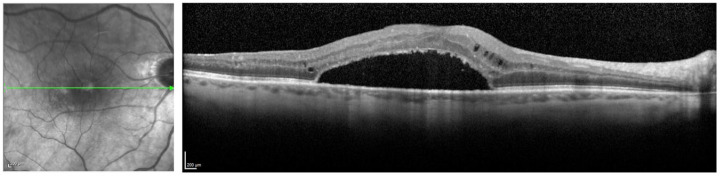
Spectral-domain optical coherence tomography of the right eye 8 months postoperatively shows minimal change in the size of loculated foveal subretinal fluid.

**Figure 6.**
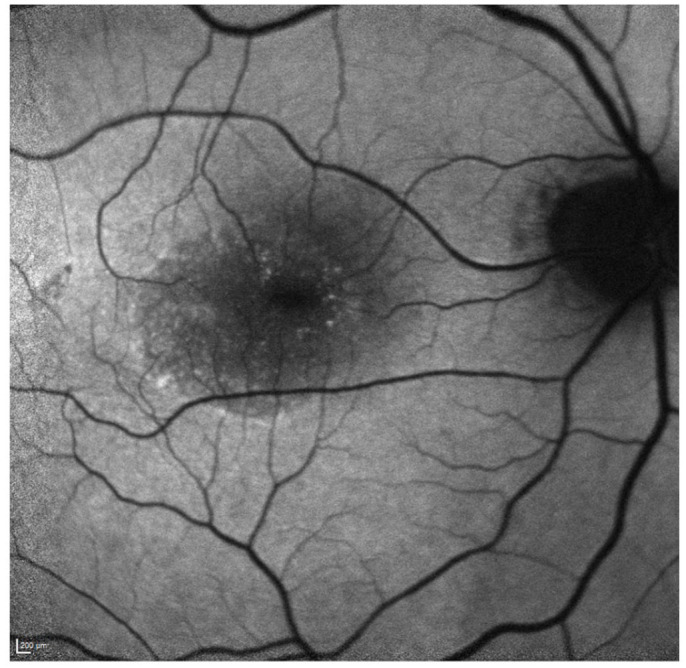
Blue fundus autofluorescence of the right eye 8 months postoperatively shows a hypoautofluorescence macula with hyperautofluorescent specks parafoveally.

Eight months after surgery, the loculated SRF persisted with minimal change in size (Figure 5). In addition, blue fundus autofluorescence showed a hyporeflective macula with hyperreflective specks parafoveally consistent with SRF (Figure 6). The BCVA in the right eye was 20/160.

## Conclusions

Persistent SRF has been described after vitrectomy surgery for FTMH, rhegmatogenous retinal detachment (RRD), and tractional RD (TRD) secondary to diabetic retinopathy (DR).^6–8^ However, to our knowledge this is the first report of the postoperative development and persistence of loculated foveal SRF after PPV for VMT.

For the treatment of symptomatic VMT that persists to the point of significant visual impairment, PPV remains the gold standard. Other management options have been investigated recently; these include enzymatic vitreolysis with intravitreal injection of ocriplasmin and pneumatic vitreolysis through intravitreal injection of gas. Recent safety concerns have tempered interest in both approaches.^9–11^

Morphologic and functional improvements after PPV for VMT have been reported in numerous studies.^4,5,12^ In a study by Petrou et al^4^ of 2 of 67 eyes with VMT that had PPV, the BCVA improved by a mean of 2 Snellen lines after surgery. Similarly, in a study by Yang et al,^5^ 17 (77%) of 22 eyes that had PPV for VMT also had an improvement in VA of at least 2 lines in addition to a significant improvement in symptoms. Furthermore, a review of OCT data found that the anatomic improvement of retinal distortion after PPV accompanies improvements in VA independent of the degree of preoperative retinal distortion.^13^

Complications of PPV for VMT include development of an RD, FTMH, ERM, CME, and cataract. In a meta-analysis of the safety profile of vitrectomy for VMT, 4.56% of cases developed an RD and 1.44% developed an FTMH.^14^ Notably, the development of persistent SRF after PPV for VMT has not been reported in the literature.

During elevation of the hyaloid membrane in our case, the development of a pocket of foveal SRF was suspected. Intraoperative OCT was not available to confirm. However, follow-up SD-OCT showed the persistence of the loculated SRF with minimal change 2 months after surgery. The persistence of SRF has been traditionally described after PPV for RRD, TRD secondary to DR, and an FTMH with RD. Unlike in VMT, the RDs and FTMHs present with SRF are the result of breaks in the retina, allowing vitreous to flow between the RPE and neurosensory retina. After surgical repair of these retinal disruptions, the RPE normally transports fluid from the subretinal space to the choroidal blood vessels, returning the subretinal architecture to normal.

Although the exact etiology of the persistence of SRF after RD repair continues to be a topic of investigation, several authors have postulated that a dysfunctional RPE, such as seen in high myopia, could be contributory.^15,16^ Indeed, high myopia has been identified as an independent risk factor for persistent loculated SRF after RRD.^15^ However, the patient in our case had low myopia with only a slightly elongated AL; thus, high myopia was less likely to be the cause of the RPE dysfunction. If SRF persists postoperatively, it can delay the improvement in BCVA for 6 to 12 months after surgery for RDs and FTMHs.^8,17^ However, the majority of studies found that the presence of persistent SRF had no overall effect on final VA after resolution in patients who have had PPV repair of FTMHs, RRDs, and diabetic tractional retinopathy.^6–8,17^

Although persistent SRF has not been reported after PPV for VMT, a case of persistent SRF in a patient with VMT treated by enzymatic vitreolysis using ocriplasmin and a case in which pneumatic vitreolysis was treated using with perfluoropropane gas have been reported. Margo et al^18^ reported a 70-year-old man who developed multiple pockets of SRF in the macular region 10 weeks after intravitreal ocriplasmin injection. Over 11 months, the SRF improved but did not resolve completely. Crosson et al^19^ described the development of a focal loculated pocket of SRF inferotemporal to the fovea 1 month after perfluoropropane gas injection. This persisted for 14 months after the injection, after which it resolved spontaneously.^1^ Similar to our patient, both cases had no sign of a retinal break or MH on fundus examination or OCT imaging. Further consistent with the findings by Margo et al,^18^ our patient’s VA was consistently worse after treatment with no improvement in or resolution of the SRF up to 8 months after surgery.

The pathophysiology of SRF in our case is unknown. In the past, authors who have reported persistent SRF after vitrectomy for FTMHs have suggested that the presence of microholes represents a gap in the photoreceptor layer, allowing for the persistence of focal SRF pockets.^6^ Indeed, the presence of a small MH of either idiopathic or iatrogenic origin was considered in this case but was ruled out after careful examination and highly sensitive SD-OCT imaging preoperatively and postoperatively. Furthermore, an important role of the RPE is phagocytic transportation of fluid, protein, and ions from the subretinal space to the choroidal blood vessels.^20^ Thus, hypotheses about persistent SRF have included dysfunction of the RPE leading to a focal area of SRF that cannot be resorbed by the RPE.^16^ Several studies suggest that a dysfunctional RPE seen in high myopia could also contribute to persistent SRF.^15,16^ In our mildly myopic patient, it is possible that focal dysfunction of the RPE led to the buildup of fluid in the subretinal space. However, further cases are required to determine the composition and origin of SRF that develops after PPV performed for VMT pathology.

In conclusion, we report the first case to our knowledge of persistent loculated foveal SRF after PPV for symptomatic VMT. Although persistent loculated SRF has been reported after vitrectomy surgery for RRD in patients with high myopia and for TRD in patients with diabetes, it has not yet been reported in the setting of VMT. Although SRF has been reported as a rare complication of other forms of treatment for VMT, including pneumatic vitreolysis and enzymatic vitreolysis, it has not been described with regard to PPV. Future studies outlining the microscopic characteristics, preoperative risk factors, and long-term course of persistent SRF in this patient population are needed to inform management decisions and patient counseling for this rare complication.

## Supplemental Material

sj-png-1-vrd-10.1177_24741264231176137 – Supplemental material for Pars Plana Vitrectomy for Vitreomacular Traction Resulting in Persistent Postoperative Loculated Foveal Subretinal Fluid Click here for additional data file. Supplemental material, sj-png-1-vrd-10.1177_24741264231176137 for Pars Plana Vitrectomy for Vitreomacular Traction Resulting in Persistent Postoperative Loculated Foveal Subretinal Fluid by Darcie S. Wilson, Ashlyn M. Pinto and R. Rishi Gupta in Journal of VitreoRetinal Diseases
