# Supplementary figures and images for: Pars Plana Vitrectomy for Vitreomacular Traction Resulting in Persistent Postoperative Loculated Foveal Subretinal Fluid
Source: J Vitreoretin Dis. 2023 Jun 6;7(6):552–6. doi: 10.1177/24741264231176137 (PMC10649448; doi:10.1177/24741264231176137)

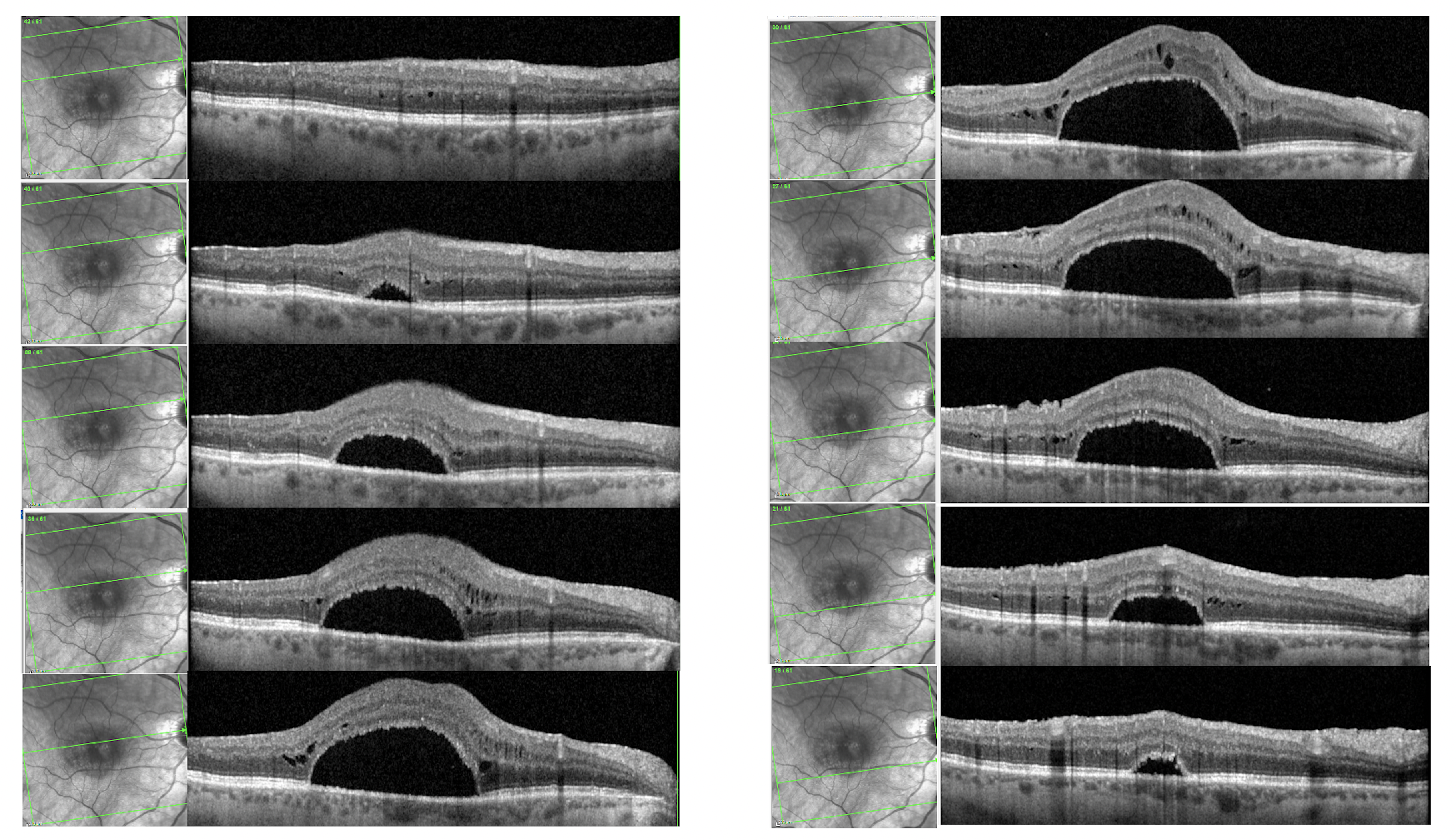

Supplement: sj-png-1-vrd-10.1177_24741264231176137 – Supplemental material for Pars Plana Vitrectomy for Vitreomacular Traction Resulting in Persistent Postoperative Loculated Foveal Subretinal Fluid [file sj-png-1-vrd-10.1177_24741264231176137.png]
